# Emotion Detection Deficits and Decreased Empathy in Patients with Alzheimer’s Disease and Parkinson’s Disease Affect Caregiver Mood and Burden

**DOI:** 10.3389/fnagi.2018.00120

**Published:** 2018-04-24

**Authors:** Maria Martinez, Namita Multani, Cassandra J. Anor, Karen Misquitta, David F. Tang-Wai, Ron Keren, Susan Fox, Anthony E. Lang, Connie Marras, Maria C. Tartaglia

**Affiliations:** ^1^Division of Neurology, Krembil Neuroscience Centre, Toronto Western Hospital, University Health Network Memory Clinic, Toronto, ON, Canada; ^2^Tanz Centre for Research in Neurodegenerative Diseases, University of Toronto, Toronto, ON, Canada; ^3^Department of Psychiatry, University Health Network, Toronto, ON, Canada; ^4^Movement Disorders Clinic and the Edmond J. Safra Program in Parkinson’s Disease, University Health Network, University of Toronto, Toronto, ON, Canada

**Keywords:** Alzheimer’s disease, Parkinson’s disease, social cognition, empathy, emotion recognition, neuropsychiatric symptoms, caregiver burden

## Abstract

**Background**: Changes in social cognition occur in patients with Alzheimer’s disease (AD) and Parkinson’s disease (PD) and can be caused by several factors, including emotion recognition deficits and neuropsychiatric symptoms (NPS). The aims of this study were to investigate: (1) group differences on emotion detection between patients diagnosed with AD or PD and their respective caregivers; (2) the association of emotion detection with empathetic ability and NPS in individuals with AD or PD; (3) caregivers’ depression and perceived burden in relation to patients’ ability to detect emotions, empathize with others, presence of NPS; and (4) caregiver’s awareness of emotion detection deficits in patients with AD or Parkinson.

**Methods**: In this study, patients with probable AD (*N* = 25) or PD (*N* = 17), and their caregivers (*N* = 42), performed an emotion detection task (The Awareness of Social Inference Test—Emotion Evaluation Test, TASIT-EET). Patients underwent cognitive assessment, using the Behavioral Neurology Assessment (BNA). In addition, caregivers completed questionnaires to measure empathy (Interpersonal Reactivity Index, IRI) and NPS (Neuropsychiatric Inventory, NPI) in patients and self-reported on depression (Geriatric Depression Scale, GDS) and burden (Zarit Burden Interview, ZBI). Caregivers were also interviewed to measure dementia severity (Clinical Dementia Rating (CDR) Scale) in patients.

**Results**: The results suggest that individuals with AD and PD are significantly worse at recognizing emotions than their caregivers. Moreover, caregivers failed to recognize patients’ emotion recognition deficits and this was associated with increased caregiver burden and depression. Patients’ emotion recognition deficits, decreased empathy and NPS were also related to caregiver burden and depression.

**Conclusions**: Changes in emotion detection and empathy in individuals with AD and PD has implications for caregiver burden and depression and may be amenable to interventions with both patients and caregivers.

## Introduction

Caregiving for people with dementia has become an issue of international importance. Dementia due to Alzheimer’s disease (AD) or Parkinson’s disease (PD) is accompanied by cognitive deficits in multiple domains, such as executive function, attention, memory and visuospatial function (Graham et al., 2004; Verbaan et al., 2007). Behavioral changes, which include neuropsychiatric symptoms (NPS) such as depression, apathy, anxiety and agitation are also prominent in individuals with dementia (Mega et al., 1996). These NPS, particularly apathy, are associated with severity of cognitive dysfunction. Furthermore, certain NPS such as irritability and aberrant motor behavior are related to high caregiver burden and increased institutionalization (Zhao et al., 2016). The impact for caregivers in managing these behavioral symptoms is significant and more difficult than managing their cognitive impairment (Victoroff et al., 1998). Moreover, caring for people with dementia negatively impacts caregiver’s health, such as depression (Hooker et al., 2002; Etters et al., 2008).

In addition to NPS, social cognition changes are reported in individuals with dementia (Snowden et al., 2003; Poveda et al., 2017). Social cognition allows individuals to partake in social situations by enabling them to perceive and recognize the thoughts, emotions, and behaviors of others (Shany-Ur and Rankin, 2011). An intact association between implicit and explicit cognitive functions are required in order to successfully decipher and interact with the social environment around us. People with dementia may display increasing difficulties in understanding social cues or recognizing emotions (Phillips et al., 2010). For instance, individuals with AD have trouble adapting to change, unconcerned with others’ feelings and are unable to control emotions. These social cognition changes are independent of cognitive dysfunction and increase over time (Cosentino et al., 2014).

Individuals diagnosed with PD also experience NPS throughout the course of the disease, particularly depression, anxiety, apathy and psychosis (Aarsland et al., 2009). Behavioral changes, such as impulsivity and compulsivity are also present in a subset of individuals diagnosed with PD and are often associated with poor quality of life (Averbeck et al., 2014). These are often not reported due to either lack of insight or embarrassment during the earlier stages of the disease (Averbeck et al., 2014). Furthermore, impulsivity in individuals with PD has in one study, been related to their inability to recognize emotions (Averbeck et al., 2014).

Individuals diagnosed with AD or PD also demonstrate emotion processing deficits, independent of their cognitive status (Gray and Tickle-Degnen, 2010; Klein-Koerkamp et al., 2012; Kumfor et al., 2014). Emotional expressions play a significant role in communication and are one of the most important aspects of social cognition (Blair, 2003; Torres et al., 2015). Emotion processing deficit in PD is often seen across different stimulus modalities, and these individuals are particularly impaired in recognizing affective prosody (Schröder et al., 2006; Paulmann and Pell, 2010). Furthermore, emotion processing deficit is independent of depression and visuospatial function, which is apparent in this group (Gray and Tickle-Degnen, 2010). Emotion recognition impairment can lead to severe consequences, such as failure to modify behavior and socialize (Blair, 2003). This can lead to breakdown in communication, increased conflict in their relationship, and caregiver burden (Orange, 1991; Richter et al., 1995). In fact, caregivers report that individuals with AD greatly depend on them and this dependence is associated with social cognition changes (Cosentino et al., 2014). However, it is unclear whether caregivers are aware of emotion detection deficits in patients with dementia.

The relationship among a patient’s ability to empathize, their emotion detection ability and caregiver’s mood has not been studied extensively. Research into the role of social cognition in neurodegenerative conditions and its impact on caregiver burden has mostly focused on patients with Frontotemporal dementia (FTD) and, only recently, in AD (Phillips et al., 2010; Shany-Ur and Rankin, 2011).

Currently, there is a dearth of studies examining the relationship between emotion detection in patients with AD or PD and their caregiver’s mood. The aims of this study are to investigate: (1) group differences in emotion detection between AD or PD patients and their respective caregivers; (2) the association of emotion detection with empathetic ability and NPS in individuals with AD or PD; (3) caregivers’ depression and perceived burden in relation to patients’ ability to detect emotions, empathize with others, presence of NPS; and (4) caregiver’s awareness of emotion detection deficits in patients with AD or PD. We hypothesized that in individuals with AD or PD who had decreased emotion detection and decreased empathy, their caregivers would have lower mood and increased burden. The investigation of changes in emotion detection and empathy has implications for caregivers and could lead to the development of effective caregiver interventions.

## Materials and Methods

### Study Participants

Forty-two patients with a clinical diagnosis of AD (*N* = 25) according to McKhann (2011) criteria or PD (*N* = 17) using the PD Society Brain Bank Clinical Diagnostic Criteria and their respective caregiver (any adult caring for the person with AD or PD) were recruited at University Health Network’s Memory Clinic and Movement Disorder’s Clinic in Toronto (Hughes et al., 1992; McKhann, 2011). All patients and their caregivers spoke and understood English. Patients and caregivers were excluded if they had a history of another neurological disorder, psychiatric disorder, visual and auditory deficits beyond requiring correction with eyeglasses or contact lens, and hearing aids. This study was carried out in accordance with the recommendations of Research Ethics Board of University Health Network with written informed consent from all subjects. All subjects gave written informed consent in accordance with the Declaration of Helsinki. The protocol was approved by the Research Ethics Board of University Health Network.

### Measures

All patients and caregivers completed The Awareness of Social Inference Test-Emotion Evaluation Test (TASIT-EET; McDonald et al., 2003). In addition, caregivers filled out an Interpersonal Reactivity Index (IRI), Neuropsychiatric Inventory (NPI), Geriatric Depression Scale (GDS), Zarit Burden Interview (ZBI) and were interviewed on the Clinical Dementia Rating Scale (CDR; Zarit et al., 1980; Yesavage et al., 1982; Davis, 1983; Morris, 1993; Cummings et al., 1994). All patients completed the revised Behavioral Neurology Assessment (BNA; Darvesh et al., 2005). For PD subjects, assessments were all performed in the ON medication state.

#### Emotion Detection

The TASIT-EET examines emotion recognition and consists of several short video clips, enacted by professional actors demonstrating seven emotions (happy, surprised, sad, angry, anxious, disgusted and neutral; McDonald et al., 2003). After each video clip, participants were instructed to select the most appropriate response (emotion) from the seven emotions (forced-choice task) shown to them on the screen. Patients and caregivers were tested separately in a quiet room. No feedback was provided after each clip. In addition, caregivers were asked to predict the patient’s response after each clip. Lower scores on this test indicate greater impairment in emotion recognition. A caregiver accuracy score was computed by subtracting the patient’s actual TASIT-EET score and the caregiver’s predicted TASIT-EET score.

#### Empathy

The IRI is a well-established 28 item self-report questionnaire on a five point Likert scale. The scale measures both the cognitive and emotional aspects of empathy. The cognitive aspects include Perspective Taking (PT) and Fantasy (F) subscales. PT involves imagining another’s perspective, whereas the F subscale assesses one’s ability to empathize for fictional characters. The emotional aspects of empathy include Empathic Concern (EC), which assesses the concern for another’s distress and Personal Distress (PersDis), a measure of personalized reactive distress (Davis, 1983). The caregivers are able to complete the IRI to assess empathy both effectively and reliably in dementia patients (Rankin et al., 2006; Sollberger et al., 2014).

#### Neuropsychiatric Symptoms

The NPI was administered to caregivers through a structured interview and is used to assess NPS in individuals with dementia. The NPI assesses the presence of the following NPS over the period of 4 weeks: delusions, hallucinations, agitation, depression/dysphoria, anxiety, euphoria/elation, apathy/indifference, disinhibition, irritability/lability, aberrant motor behavior, sleep and appetite and eating disorder (Cummings et al., 1994). In the presence of symptoms, caregivers are asked about the frequency (4-point scale) and severity (3-point scale) of these symptoms and on caregiver distress. A total score for each symptom is obtained by multiplying the frequency and severity score. Lastly, caregiver distress is rated on a 6-point scale (Cummings et al., 1994; Kaufer et al., 1998). For this current study, we used four neuropsychiatric syndrome groups: psychotic (hallucinations and delusions), affective (depression and anxiety), hyperactivity (agitation, euphoria, irritability, disinhibition and aberrant motor behavior) and apathetic (apathy and eating abnormalities; Aalten et al., 2007).

#### Depression

The GDS was administered to all caregivers. It is a 15 item (yes or no response) self-reported questionnaire used to identify depression in older adults. A higher score indicating greater symptoms of depression (Yesavage et al., 1982).

#### Caregiver Burden

The caregivers filled out a 22-item ZBI questionnaire to evaluate the amount of stress experienced by them due to the person’s dementia. Each item is rated on a 5-point scale, and a total score is calculated by taking the sum of individual statements. A higher score on the ZBI indicates increased burden (Zarit et al., 1980).

#### Staging of Dementia Severity

The CDR is used as a staging scale to assess the severity of AD through a semi-structured interview with a caregiver. It rates subject’s cognitive ability on six separate domains (memory, orientation, judgment and problem solving, community affairs, home and hobbies and personal care) using a five-point scale (Morris, 1993). The CDR sum of boxes (CDR-SoB) was used in the analysis.

#### Cognitive Assessment

The revised BNA is a brief cognitive assessment that takes about 45 min to administer and covers major cognitive domains. It consists of 24 subtests, which are categorized into six subdomains (attention, memory, language, visuospatial function, executive function and praxis). In addition to individual domain scores, a total score is also calculated. For the purpose of this article, only the BNA cumulative percentage (BNA cumulative %) was used (Darvesh et al., 2005).

### Statistical Analysis

The SPSS Statistics 23 software was used to conduct the statistical analysis. Group comparisons on age, gender, GDS, IRI (PT and EC sub-scores), CDR-SoB and revised BNA cumulative percentage were carried out using the independent samples *t*-test. A chi-square was conducted to look for gender differences. We performed an analysis of covariance (ANCOVA) to examine for TASIT-EET performance differences between the two patient groups, while adjusting for covariates (age, gender and CDR-SoB). ANCOVA was also carried out to assess TASIT-EET performance in AD caregivers and PD caregivers, while adjusting for covariates (age and gender).

One-way ANOVA was performed for each patient group (AD and PD) to assess caregiver’s accuracy in predicting the patient’s response on TASIT-EET. TASIT-EET performance differences were measured amongst the three groups (patient, caregiver and caregiver-predicted-patient-response) and a Tukey *post hoc* test was performed to analyze individual group differences.

A one-tailed Pearson correlation was performed between patients’ TASIT-EET performance, and BNA cumulative %, IRI-PT, IRI-EC and NPI total score. Next, to determine factors that correlated with caregiver burden and depression, a one-tailed Pearson correlation was carried out between ZBI and GDS, and patients’ TASIT-EET score, TASIT-EET accuracy score (the caregiver’s ability to accurately predict patient’s TASIT response; accuracy = patient response − caregiver-predicted-patient-response), IRI-PT, IRI-EC, NPI-apathetic (NPI-Ap), NPI-affective (NPI-Af), NPI-hyperactivity (NPI-H), NPI-psychotic (NPI-P) and BNA cumulative %. A TASIT-EET accuracy score closer to zero would represent greater accuracy, whereas a score further away from zero (positive or negative) would represent less accuracy in caregiver’s ability to predict the patient’s response. The correlation was performed for all subjects, AD subgroup and PD subgroup. Due to the small sample size, Spearman correlation was performed for the PD group. Bonferroni correction was applied to correct for multiple comparisons.

## Results

### Study Participants and Characteristics

Patients with very mild to moderate dementia due to AD (*N* = 25) and PD (*N* = 17), ranging from none to mild dementia, differed in terms of gender, CDR-SoB and revised BNA total score (Table 1). The two patient groups did not differ significantly in terms of age, IRI-PT, IRI-EC and NPI total score. There was also no significant difference between patients with AD or PD on TASIT-EET score (*F*_(1,33)_ = 1.13, *p* = 0.295), while adjusting for age, gender and CDR-SoB (Table 1).

**Table 1:** Patient demographics, clinical dementia rating (CDR) sum of boxes, cognitive score, the awareness of social inference test-emotion evaluation test (TASIT-EET) score, interpersonal reactivity index (IRI) sub-scores and neuropsychiatric inventory (NPI) total score.

	AD (*N* = 25)	PD (*N* = 17)	*p*-value
Age	73.2 ± 10.7 years	69.35 ± 8.2 years	0.219
Gender (M, F)	10, 15*	15, 2*	0.003*
Revised BNA cumulative %	54.45 (± 17.4)*	76.23 (± 13.6)*	<0.05*
CDR-SoB*	4.48 ± 2.3*	2.79 ± 2.0*	0.02*
TASIT-EET score	7.47 ± 2.2	8.88 ± 2.2	0.295
IRI perspective taking (PT)	19.58 ± 5.8	21.00 ± 7.8	0.514
IRI empathetic concern (EC)	25.16 ± 5.6	26.65 ± 7.2	0.454
NPI total score	9.92 ± 10.5	17.38 ± 22.6	0.159

*p < 0.05.

Patients in both groups were taking the following medication: acetylcholinesterase inhibitors, antihypertensive agents, anti-inflammatory, anticoagulants, antidepressants, anxiolytics, cholesterol-lowering agents, gastrointestinal agents, supplements and thyroid medication. In addition, some individuals diagnosed with AD were also taking asthma medication, bone-building supplements, muscle relaxants and medication for prostate enlargement. Whereas, some individuals in the PD group were on anticonvulsants, central nervous system stimulants, corticosteroids, dopamine agonists, erectile dysfunction, heart medication, hormone replacement therapy, hypoglycemic agents and sedatives.

Comparing caregivers of individuals diagnosed with AD or PD, there was no significant difference in age, gender, TASIT-EET score, GDS total score, ZBI total score and NPI distress score (Table 2).

**Table 2:** Caregiver demographics, TASIT-EET score, geriatric depression scale (GDS) total score, Zarit burden interview (ZBI) total score and neuropsychiatric inventory (NPI) distress score.

	AD (*N* = 25)	PD (*N* = 17)	*p*-value
Age	62.05 ± 13.3 years	67.35 ± 9.4 years	0.171
Gender (M, F)	8, 14	3, 14	0.288
TASIT-EET score	10.59 ± 2.0	11.00 ± 1.8	0.466
GDS	2.93 ± 3.7	3.65 ± 3.5	0.539
ZBI	30.90 ± 13.7	29.9 ± 19.3	0.851
NPI distress	6.20 ± 5.3	9.81 ± 11.5	0.181

### Emotion Detection in Patients and Caregivers

**Figure 1:**
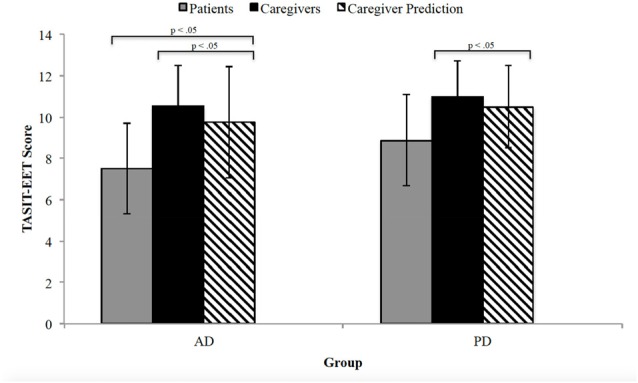
The Awareness of Social Inference Test-Emotion Evaluation Test (TASIT-EET) average score for patients (gray), caregivers (black) and caregiver perspective of patient’s response (black diagonal lines). The error bars represent standard deviation. AD, Alzheimer’s disease; PD, Parkinson’s disease.

A one-way ANOVA comparing TASIT-EET scores revealed statistically significant differences among AD patients, caregivers and caregivers’ patient predicted score, *F*_(2,70)_ = 10.94, *p* < 0.0005. Caregivers of AD patients (10.52 ± 2.0) scored significantly higher on TASIT-EET than AD patients (7.52 ± 2.2), scoring three points higher (95% CI, 1.41–4.59, *p* < 0.0005). Moreover, the AD caregivers’ patient predicted TASIT-EET score (9.76 ± 2.7) was also significantly greater than patients’ actual performance (mean difference = 2.24, 95% CI 0.65–3.83, *p* < 0.05). The three PD groups (patients, caregivers and caregiver’s predicted response) also significantly differed from one another, *F*_(2, 47)_ = 5.20, *p* < 0.05. This difference was primarily due to a difference in the TASIT-EET score between patients (8.88 ± 2.2) and their caregivers (11.00 ± 1.8); mean difference = 2.12, 95% CI 0.46–3.78, *p* < 0.05. The predicted TASIT-EET score (10.5 ± 2.2) was also greater than the patient’s actual score but was not significant (mean difference = 1.62, 95% CI −0.07 to 3.30, *p* = 0.062). Figure 1 displays the average score for patients, caregivers, and the caregiver-perspective of the patient’s response.

### Emotion Detection Correlates in AD and PD

The TASIT-EET score was significantly associated with BNA cumulative %, IRI-EC and IRI-PT. However, as expected, only the BNA cumulative % survived correction for multiple comparisons since there is an overlap between loss of empathy and TASIT-EET score (Table 3). No significant correlation was evident for the AD group, whereas, a significant positive correlation was seen in the PD group between the patient’s TASIT-EET, and BNA cumulative % and IRI-PT, which survived correction for multiple comparisons (Table 3).

**Table 3:** Correlation between the patients’ TASIT Emotion Evaluation Test and their Interpersonal Reactivity Index sub-scores.

	All subjects TASIT-EET (*N* = 42)		AD patients TASIT-EET (*N* = 25)		PD patients TASIT-EET (*N* = 17)	
	*r*	*p*	*r*	*p*	*r*	*p*
Revised BNA cumulative %	0.628**	<0.0005	0.533*	0.006	0.696**	0.001
IRI-EC	0.287*	0.036	0.163	0.229	0.394	0.059
IRI-PT	0.361*	0.013	−0.027	0.452	0.789**	<0.0005
NPI total score	−0.112	0.249	−0.037	0.433	−0.327	0.108

*p < 0.05, **p < 0.003.

### Caregiver Depression and Burden Correlates

A significant correlation was found between caregiver ZBI and patient TASIT-EET, IRI-PT, IRI-EC, NPI-Af, NPI-Ap, NPI-H and BNA cumulative % for both AD and PD patients (Table 4). There was also a positive association between NPI-P and caregivers’ ZBI but this did not withstand correction for multiple comparisons. When examining the AD and the PD group separately, the AD group demonstrated a significant correlation between the caregivers’ ZBI and AD patient IRI-EC, IRI-PT and NPI-Ap. In the PD group, on the other hand, there was a significant association, which survived correction for multiple comparisons, between ZBI, and NPI-Af, NPI-H and BNA cumulative % (Table 4).

**Table 4:** Association between caregiver’s Zarit Burden Inventory score, and Interpersonal Reactivity Index subscales and Neuropsychiatric Inventory subscales.

	All Caregivers ZBI (*N* = 42)		AD Caregiver ZBI (*N* = 25)		PD Caregiver ZBI (*N* = 17)	
	*r*	*p*	*r*	*p*	*r*	*p*
Revised BNA cumulative %	−0.446**	0.002	−0.378*	0.041	−0.67**	0.002
TASIT-EET	−0.325*	0.02	−0.268	0.108	−0.389	0.061
IRI-EC	−0.464**	0.001	−0.537**	0.003	−0.354	0.082
IRI-PT	−0.612**	<0.0005	−0.641**	<0.0005	−0.535*	0.016
NPI-Af	0.483**	0.001	0.495	0.006	0.805**	<0.0005
NPI-Ap	0.465**	0.001	0.548**	0.002	0.476*	0.031
NPI-H	0.438**	0.002	0.237	0.127	0.759**	<0.0005
NPI-P	0.292*	0.032	0.402*	0.023	−0.088	0.373

*p < 0.05, **p < 0.003.

A significant association was also found between caregiver GDS and all patients’ TASIT-EET score, IRI, NPI sub-scores and BNA cumulative %. However, only the relationship between caregiver GDS with NPI-Ap, NPI-H and NPI-P withstood correction for multiple comparisons. The AD and PD groups were also examined separately and in the AD group, significant correlations were found which survived correction for multiple comparisons for caregiver GDS with IRI-EC and NPI-P. Whereas, in the PD group, the caregiver’s GDS demonstrated a significant relationship with NPI-Af and NPI-Ap sub-score, but these did not survive multiple comparison correction (Table 5).

**Table 5:** Association between caregiver’s Geriatric Depression Score, and Interpersonal Reactivity Index subscales and Neuropsychiatric Inventory subscales.

	All Caregiver’s GDS (*N* = 42)		AD Caregiver’s GDS (*N* = 25)		PD Caregiver’s GDS (*N* = 17)	
	*r*	*p*	*r*	*p*	*r*	*p*
Revised BNA cumulative %	−0.294*	0.038	−0.4*	0.04	−0.598*	0.006
TASIT-EET	−0.291*	0.038	−0.315	0.082	−0.295	0.125
IRI-EC	−0.397*	0.008	−0.551**	0.003	−0.171	0.255
IRI-PT	−0.42*	0.004	−0.465*	0.013	−0.205	0.223
NPI-Af	0.381*	0.008	0.161	0.232	0.593*	0.008
NPI-Ap	0.434**	0.003	0.396*	0.031	0.438*	0.045
NPI-H	0.457**	0.002	0.334	0.059	0.178	0.255
NPI-P	0.638**	<0.0005	0.748**	<0.0005	0.106	0.349

*p < 0.05, **p < 0.003.

**Figure 2:**
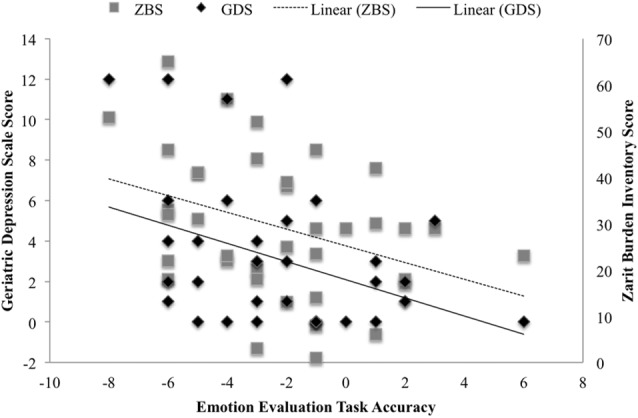
Association between the TASIT-EET accuracy score, and caregiver Geriatric Depression Scale (GDS) score and caregiver Zarit Burden Interview (ZBI) score.

Lastly, there was a statistically significant negative association between the TASIT accuracy score (caregiver’s ability to accurately predict patient’s response), and ZBI (*r* = −0.331, *p* < 0.05) and GDS (*r* = −0.387, *p* < 0.01; Figure 2).

## Discussion

This study investigated the social cognition changes in patients with AD or PD and its effect on their caregivers. Our results indicate that patients with AD or PD do not differ from one another on emotion recognition and empathy. However, patients with AD or PD have a decreased ability to detect emotions compared to their caregivers. These findings corroborate previous research which demonstrates that compared to their partners, patients with AD are impaired at recognizing emotions (Poveda et al., 2017). Individuals with AD were impaired at recognizing all emotions, with the exception of happiness (Poveda et al., 2017). Furthermore, based on our findings, these deficits in patients with AD are associated with caregiver burden. In regards to the NPS, although the PD group demonstrates greater and wider range of NPS than the AD group, this difference was not significant. The caregivers of these patients also do not differ from one another in relation to their emotion detection ability, depression, perceived burden and NPS distress.

Current literature suggests that individuals with PD show affective prosody and broad facial emotion recognition impairment (Ibarretxe-Bilbao et al., 2009; Paulmann and Pell, 2010; Baggio et al., 2012). However, there is contradicting evidence where patients with PD did not display any emotion detection deficits (Wabnegger et al., 2015; Ille et al., 2016). Assogna et al. (2008) attributes this inconsistency to a number of different factors, such as differences in emotion assessment, perception deficits, cognitive impairment, behavioral symptoms, dopamine replacement therapy and illness severity. The patients from the Wabnegger et al. (2015) and Ille et al. (2016) group had no cognitive deficits, whereas, PD patients in the current study were mildly cognitively impaired.

Although PD patients on dopamine replacement therapy perform significantly better on recognizing disgust than unmedicated patients, the medicated group is poor at recognizing emotions compared to healthy controls (Sprengelmeyer et al., 2003). Therefore, dopamine replacement therapy alone cannot explain emotion recognition impairment in this PD group. Moreover, emotion recognition in PD is unrelated to basic face recognition and motor impairment (Clark et al., 2008). However, based on our findings, the ability to accurately detect emotions was related to the PD patients’ cognitive function and their ability to perceive others’ point of view. This was not significant for the AD patients, suggesting that emotion detection impairment may be related to differences in neuroanatomical regions affected in each disease (Hughes et al., 1992; McKhann, 2011). However, there is evidence that general cognitive deficits can account for some variance in regards to emotion perception. Successful emotion recognition may require intact executive functioning skills and decision-making skills (Dujardin et al., 2004; Phillips et al., 2010; Poveda et al., 2017). The emotion evaluation stimuli for the current study consisted of dynamic emotion stimuli (facial expression, words and tone) whereas previous studies utilized static emotional expressions. We speculate that cognitive deficits may play a role in emotion recognition impairment, as dynamic emotion stimuli require participants to integrate information from multiple modalities.

Previous studies report a relationship between an indifference to interpersonal relationships and recognition of static facial images of emotion in patients with AD (Shimokawa et al., 2001). It is clear from our results that emotion detection and certain aspects of empathy are related, evident when both patient groups are combined. Interestingly we found a significant correlation between emotion detection and PT in PD patients but no relationship was found in the AD patients. The differences could be related to the level of impairment in the AD group that may have a greater impact on emotion detection than loss of empathy. These emotion detection deficits were also related to caregiver burden and depression, which has also been previously reported in AD (Miller et al., 2013).

There is evidence that emotion recognition impairment is associated with focal atrophy in cortical, as well as subcortical regions (Rankin et al., 2006; Lévêque, 2014). Therefore, disease-specific atrophy in different neurodegenerative diseases may cause specific patterns of emotion recognition deficits in these diseases. For instance, the amygdala is involved in recognition of emotions from facial expressions, particularly negative emotions, whereas, the frontal region is implicated in evaluation of positive emotions (Adolphs, 2001; Rosen et al., 2004). Within the PD group, static facial emotion recognition is associated with bilateral orbitofrontal cortex atrophy (Ibarretxe-Bilbao et al., 2009). Moreover, subcortical regions, such as the nucleus accumbens, are also connected to limbic system and are involved in regulating emotions (Lévêque, 2014). Functional imaging studies suggest that basal ganglia regions, putamen and the head of the caudate, are activated during affective prosody processing (Kotz et al., 2003). Hence, several neural structures, some of which are involved in PD and AD progression, may contribute towards emotion recognition deficits in AD and PD patients.

The findings of the current study also indicate that both caregiver burden and depression in caregivers of patients with AD or PD are also associated with social cognition changes in these patient populations. Overall, increased apathy and hyperactivity NPS in patients are associated with increased caregiver burden and depression. However, patients’ decreased emotion detection ability, PT ability, EC, and increased affective NPS are associated with only increased burden in their caregivers. On the other hand, increased psychotic NPS are related to depression only.

Behavioral issues in individuals with dementia has previously been associated with negative health outcomes, such as cardiovascular disease, in caregivers (Mausbach et al., 2007). Depression in dementia patients is known to be significantly associated with caregiver depression (Ornstein and Gaugler, 2012). However, findings on caregiver burden were inconsistent as the authors found that there was no specific NPS that is consistently associated with caregiver burden (Ornstein and Gaugler, 2012). For instance, some studies suggest that anger/aggression is a predictor of caregiver burden, whereas, others found sleep disturbances to be primarily associated with caregiver burden. These mixed findings may be attributed to different types of dementia. This can also help explain the differences found in our study on the relationship between NPS and caregiver burden/depression in AD and PD. Within the AD group alone, patients’ decreased EC is associated with both caregiver depression and burden. Whereas, capacity of AD patients to take on others’ point of view and apathy are related to caregiver burden alone and psychotic symptoms are associated with only depression. The PD group, however, display increased caregiver burden in relation to affective and hyperactivity NPS.

A novel and important finding of this study is that caregivers of AD and PD patients are poor at predicting patients’ emotion detection response. That is, when caregivers are asked to predict the patient’s insight into detecting others’ emotions, overall, they overestimate the patients’ ability to accurately detect emotions. In fact, it appears that caregivers believe that the patients will give a similar response to their own. Furthermore, the caregivers’ inability to accurately predict patients’ emotion recognition response is associated with increased burden and depression. There may be misinterpretation of a patient’s lack of appropriate reaction to caregiver’s emotion as not being caring when in fact the patient is unable to recognize emotions. When caregivers feel a lack of consideration for their emotions, they can feel unappreciated, leading to low mood and burnout. Education and behavioral skills training for caregivers who are caring for patients with dementia with challenging behaviors has led to reduced burden in caregivers (Schulz and Martire, 2004). Therefore, similar strategies for emotion recognition deficits may lead to decreased burden in caregivers. For instance, having caregivers reinforce an emotion by stating it plainly without assuming that the patient can accurately detect from facial emotions or body language, may improve communication between the pair.

A possible limitation of the current study is that our sample size is small so we take caution in generalizing these results to a wider population. Another limitation is our inability to look carefully at the influence of gender on the various relationships we found. Most of the literature relating to the mood of care partners of people with dementia has focused on behavioral symptoms, where negative behavioral symptoms lead to a decline in the care partners’ mood and health (Mausbach et al., 2007; Ornstein and Gaugler, 2012; Miller et al., 2013). This study adds to our understanding that caregivers’ depressive symptoms and burden are directly related to: (a) the patient’s decreased capacity to empathize; (b) the patient’s decreased ability to take on someone else’s perspective; and (c) the caregivers lack of appreciation for emotion detection deficits in the patients. These findings demonstrate that the decreased ability to detect emotions by patients with AD and PD may negatively affect the relationship with the person most responsible for their care.

In conclusion, the results of this study suggest that patients with AD and PD have a decreased ability to detect emotion in others. In patients with PD, difficulty understanding another’s point of view is associated with their inability to accurately detect emotions. Notably, caregivers do not seem to be aware of emotion detection deficits in the patients and overestimate the patients’ abilities to detect emotions. This unawareness of an emotion detection deficit was associated with both depression and burden in the caregivers. Being a caregiver to those with dementia has been associated with negative effects on the care partners’ health and this study provides some insight into the profound challenges that changes in behavior can create for caregivers looking after a person with AD or PD (Etters et al., 2008). These behavioral changes (decreased emotion detection and decreased empathy for others) and their caregivers’ unawareness of the changes warrant further study as this may lead to interventions and therapeutic approaches that could decrease caregiver burden and improve well-being. These interventions should be aimed at: (a) providing education to caregivers to inform them that decreased emotion detection and decreased empathy can be part of the disease process; (b) increasing competence in emotion detection by providing additional cues to the patient related to emotions such as stating plainly their feelings without assuming patient can detect the emotion with facial cues or bodily language; and (c) to learn management strategies that caregivers can adapt over time. Future work is needed in examining emotion detection and empathy in other dementias.

## Author Contributions

MM conducted literature search, data analysis and wrote the manuscript. NM was involved in data collection, data analysis and manuscript editing. CJA and KM collected data and edited the manuscript. DFT-W, RK, SF, AEL, CM and MCT were involved in data interpretation and manuscript editing.

## Conflict of Interest Statement

The authors declare that the research was conducted in the absence of any commercial or financial relationships that could be construed as a potential conflict of interest.
